# Emlen funnel experiments revisited: methods update for studying compass orientation in songbirds

**DOI:** 10.1002/ece3.2383

**Published:** 2016-09-07

**Authors:** Giuseppe Bianco, Mihaela Ilieva, Clas Veibäck, Kristoffer Öfjäll, Alicja Gadomska, Gustaf Hendeby, Michael Felsberg, Fredrik Gustafsson, Susanne Åkesson

**Affiliations:** ^1^ Centre for Animal Movement Research Department of Biology Lund University Ecology Building SE‐223 62 Lund Sweden; ^2^ Institute of Biodiversity and Ecosystem Research Bulgarian Academy of Sciences 2 Gagarin street 1113 Sofia Bulgaria; ^3^ Division of Automatic Control Department of Electrical Engineering Linköping University SE‐581 83 Linköping Sweden; ^4^ Computer Vision Laboratory Department of Electrical Engineering Linköping University SE‐581 83 Linköping Sweden

**Keywords:** Computer vision, image analysis, magnetic alignment, navigation

## Abstract

1. Migratory songbirds carry an inherited capacity to migrate several thousand kilometers each year crossing continental landmasses and barriers between distant breeding sites and wintering areas. How individual songbirds manage with extreme precision to find their way is still largely unknown. The functional characteristics of biological compasses used by songbird migrants has mainly been investigated by recording the birds directed migratory activity in circular cages, so‐called Emlen funnels. This method is 50 years old and has not received major updates over the past decades. The aim of this work was to compare the results from newly developed digital methods with the established manual methods to evaluate songbird migratory activity and orientation in circular cages.
2. We performed orientation experiments using the European robin (*Erithacus rubecula*) using modified Emlen funnels equipped with thermal paper and simultaneously recorded the songbird movements from above. We evaluated and compared the results obtained with five different methods. Two methods have been commonly used in songbirds’ orientation experiments; the other three methods were developed for this study and were based either on evaluation of the thermal paper using automated image analysis, or on the analysis of videos recorded during the experiment.
3. The methods used to evaluate scratches produced by the claws of birds on the thermal papers presented some differences compared with the video analyses. These differences were caused mainly by differences in scatter, as any movement of the bird along the sloping walls of the funnel was recorded on the thermal paper, whereas video evaluations allowed us to detect single takeoff attempts by the birds and to consider only this behavior in the orientation analyses. Using computer vision, we were also able to identify and separately evaluate different behaviors that were impossible to record by the thermal paper.
4. The traditional Emlen funnel is still the most used method to investigate compass orientation in songbirds under controlled conditions. However, new numerical image analysis techniques provide a much higher level of detail of songbirds’ migratory behavior and will provide an increasing number of possibilities to evaluate and quantify specific behaviors as new algorithms will be developed.

## Introduction

Bird migration is one of the most spectacular phenomena in nature and has caught the attention of scientists for centuries. Perhaps the most challenging task of migration is for animals to find their way. Migratory songbirds rely on an endogenous migration program inherited from their parents during their first migration. In this program, duration of migration and directions to fly are encoded (“clock‐and‐compass”; e.g. Berthold 1996). For their orientation, songbirds have access to a number of biological compasses based on information from the sun, stars, and the Earth's magnetic field (e.g., Åkesson et al. 2014). Compass mechanisms have been mainly studied in caged songbird migrants (e.g., Emlen 1975; Wiltschko and Wiltschko 1991; Able 1993; Åkesson 1994; Åkesson and Bäckman 1999; Muheim et al. 2006a,b). A widely used technique to record in circular cages the migratory restlessness expressed by songbirds was described by Emlen and Emlen (1966). The method involves a funnel‐shaped cage, known as the “Emlen funnel,” where the birds’ activity is recorded as claws marks left on a paper placed on the sloping walls of the cage. In the last decades, the paper method has not been updated, apart from replacing the use of the ink and white paper of the original design with first Tipp‐Ex^®^ paper (Rabøl 1979; Beck and Wiltschko 1981), and in more recent years with thermal paper (Mouritsen et al. 2009).

The marks left on the paper were individually counted in sectors, usually on a backlight table (Wiltschko and Wiltschko 1972; Helbig 1991; Wiltschko et al. 1993). This operation can be very time‐consuming especially when birds are highly active. To limit the need of counting all scratches on a paper, counting of scratches crossing a horizontal line was introduced (Åkesson 1993, 1994). Despite this simplification, it is common to count thousands of scratches on a single paper during one‐hour tests (Åkesson 1994; Åkesson et al. 1995). To speed up the process of evaluating scratches on paper, a fast visual evaluation method was proposed by Mouritsen (1998). Both above‐mentioned methods have the disadvantage of being potentially user‐biased.

To speed up the paper evaluation procedure and overcome the uncertainty due to user experience and sensibility, a more robust method is desirable. Nowadays, computer‐based image analysis provides ready available algorithms for the quantification of several features of digital images (Abràmoff et al. 2004; Schneider et al. 2012). Instead of relying on human vision, an automated classification of the thermal paper would be a more objective way to obtain the bird orientation activity recorded in the Emlen funnel.

With any method used for evaluating the Emlen funnel paper, the sole information obtained is the mean orientation and a rough estimation of the overall activity without any temporal resolution (cf. Åkesson and Sandberg 1994; Muheim et al. 2014). However, with alternative methods, the level of migratory restlessness can be measured, not only in terms of number of jumps, but also considering intensity and degree of wing fluttering or other relevant motility information such as movement distance and jump height. Furthermore, there are others behaviors, in addition to orientation and activity that may be of interest to record during the experiments. For example, many vertebrates have been shown to align their body axis along the Earth's magnetic field (Begall et al. 2013 and reference therein). While there are different speculations about the reason for this phenomenon, the magnetic alignment in birds has only been considered as results of Emlen funnel experiments (i.e., jumps at takeoff attempts) and not measuring the body alignment like in other species (Begall et al. 2013).

Development of new methods should try to address this multitude of behaviors to provide a better understanding of the migratory activity. It is, however, not reasonable to acquire such large number of parameters with manual annotation, but by applying novel digital methods partially or completely automated.

Cages with automatic registration of restlessness activity have been utilized in the past for evaluating orientation in songbirds (Wallraff and Gelderloos 1978; Beck and Wiltschko 1981; Sandberg et al. 1988; Helbig 1991; Åkesson 1994; Åkesson and Sandberg 1994), but they did not provide more precise/accurate data than the paper evaluation method (Muheim et al. 2014). These methods used plates (eight) connected to microswitches or infrared‐light sensors providing relatively low angular resolution and were affected by calibration problems (Sandberg et al. 1988; Mouritsen and Larsen 2001; Muheim et al. 2002). More importantly, the songbirds’ magnetic sense is negatively affected by electromagnetic noise (Ritz et al. 2004; Engels et al. 2014), and thus, the use of any electromagnetic source, such as electronic devices, during experiment should be avoided. On the other hand, the use of computer vision applications is widely used in animal movement studies because it allows tracking single individuals remotely from reasonably long distances (Dell et al. 2014; Pérez‐Escudero et al. 2014). Video‐recordings of behavioral data have already been used in bird migration studies and allow addressing specific characteristics of the behavior, like head scanning or circadian flight schedules (e.g., Mouritsen et al. 2004; Coppack et al. 2008). More recently, dedicated software applied to birdcage experiments has been made freely available to the songbird research community (e.g., http://canmove.lu.se/birdoritrack) for evaluations of movement activity and orientation (Muheim et al. 2014). However, new tools come also with some limitations, and sometimes, it is challenging to assess the cost and benefit of a new method without a systematic study comparing among traditional methods and the newly developed ones.

In this study, we compared five different methods to estimate the orientation of songbirds during migratory restlessness using modified Emlen funnels. Two of the methods were based on standard manual evaluation by eye of thermal paper outlined above. A third method based on an automated image analysis of thermal paper was developed for the purpose of this study. The fourth and fifth methods were based on the analysis of the video recorded during the experiments. We selected specific cases of our dataset to exemplify the differences between the methods to track the birds’ behavior and to discuss how such differences could affect a full‐scale study on songbirds’ orientation. Based on our findings, we propose guidelines for future research on songbird orientation outlining relevant recommendations for data interpretation and future method developments.

## Experimental Procedure

**Figure 1:**
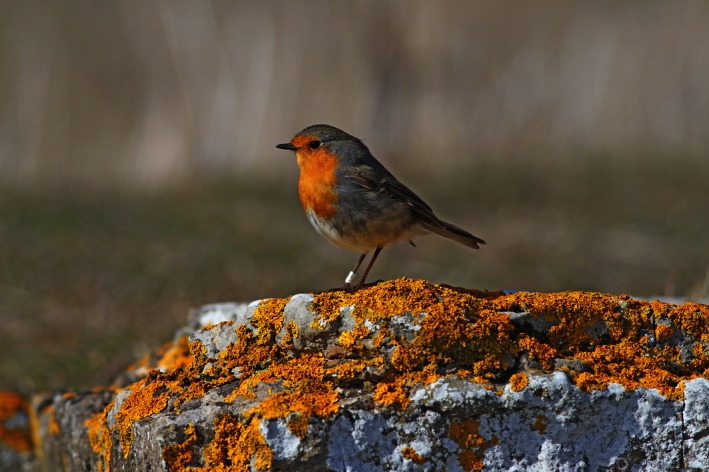
European robin (*Erithacus rubecula*) was the species used in this study to compare different methods to evaluate orientation assays performed in the Emlen funnel. Photograph by Magnus Hellström.

In our experiments, we used first‐year European robins (*Erithacus rubecula*; Fig. 1), a migratory songbird species that has been used extensively in orientation studies, including the work by Wiltschko and Wiltschko (1972), describing the function of the magnetic (inclination) compass in birds.

Experimental birds were captured with mist nets at an inland stopover site in South Sweden (55°41′N 13°26′E) during autumn migration. The birds’ activity was recorded indoors in modified Emlen funnels equipped with thermal paper (Åkesson 1994), at the Field Station run by the Department of Biology at Lund University. We performed three 1‐h assays on the same day at sunset (local time 16:30, UTC + 2 h, 1st November 2014) and after 1.5 and 3 h. In total, four birds were used for all three assays and were randomly assigned to a cage position each time they were included in experiments. Conducting three consecutive assays during the same night allowed us to capture different levels of activity and provided us with a reasonable sample size (*n* = 12) to compare methods qualitatively (Figs. S1–S5). We video‐recorded each assay from above using a D‐Link DCS‐7513 infrared‐light‐equipped network camera connected to a personal computer located in a separate house.

### Evaluations by Thermal paper

#### Method 1. Manual counting

The counting procedure of claw marks on the thermal paper followed the method outlined in Åkesson (1994). After the experiments ended, the papers were divided into 24 sectors and the scratches in each sector, passing a horizontal line with the majority of registrations, were manually counted by both an experienced and a naïve user (i.e., the latter without previous experience in bird orientation procedures). The number of scratches for each sector was represented as a circular histogram (Fig. S1) and was evaluated with standard circular statistic methods. The level of activity was given by the sum of the scratches counted for all 24 sectors (Figs. S7 and S9).

#### Method 2. Visual estimation

We estimated the claw marks left on the thermal paper according to Mouritsen (1998). In this procedure, a person visually estimates the preferred direction and two indices, one for activity and the other for concentration of the circular distribution, by looking at the claw marks left on the thermal paper lining the funnel walls when still arranged in the funnel. Both indices are defined on a 0–4 scale (for details see Mouritsen 1998). The estimated orientation angle was combined with both activity and concentration indices in a circular plot (Fig. S2). The sole activity index was also plotted for comparison with the other methods (e.g., Fig. S7).

#### Method 3. Automatic evaluation

**Figure 2:**
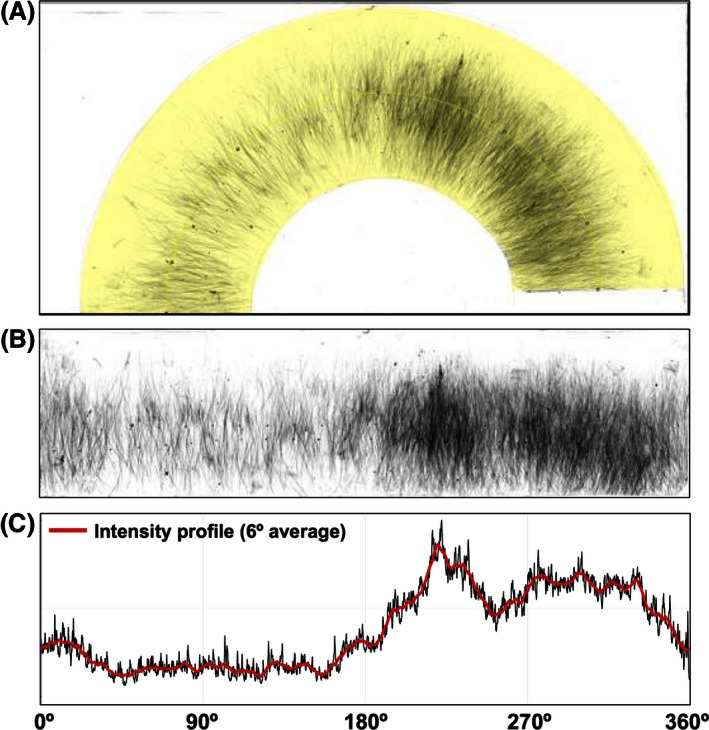
Procedure for the automatic evaluation of claw marks left on thermal paper. (A) The thermal paper is first digitalized in 8‐bit gray scale file and successively manually selected in the scanned image (shaded yellow area). (B) The selection is automatically straightened to align the claw marks along the vertical axis. (C) The image intensity profile (see text for definition) is plotted and successively smoothed with a 6° moving average filter before being used for orientation evaluation.

The procedure for the automatic evaluation of claw marks left on the thermal paper followed three main steps: (1) convert the thermal paper into a digital image, (2) geometrically transform the semicircular paper into a rectangular shape, (3) measure the vertical intensity profile of the rectangular paper across its width (Fig. 2).

First, the thermal paper was scanned in 8‐bit grayscale image at a resolution of 600 dpi using an office copier equipped with a flat scanner (Ricoh Aficio MP C2000 Tokyo, Japan). As the thermal paper was larger than the A3 maximum scanner size, the left and right side of the paper were scanned separately. Left and right images were then imported in ImageJ software version 1.49 (Abràmoff et al. 2004) and were automatically stitched together in a single image using the ImageJ *Stitching* plugin (Preibisch et al. 2009).

After the scanning, the only manual step of the procedure was the selection of the semicircular shape of the thermal paper (Fig. 2A). This was performed with a spline fitting of a 7‐point segmented selection with width equal to the paper height (i.e., 1000 pixel). The selection was then automatically straightened using the ImageJ *Straighten* plugin (Kocsis et al. 1991) to align the claw marks along the vertical axis (Fig. 2B).

To evaluate the angular distribution of claw marks, we measured the intensity profile of the straightened selection, that is the mean vertical luminosity across the horizontal direction of the image. Given the image of n \times m pixels (in the horizontal *x* and vertical *y* directions, respectively), the mean vertical luminosity *L* for each *i = 1, …, n* was calculated as the mean of the pixel luminosity *p* for all the vertical aligned pixels *j = 1, …, m* as follows:

$$L\left(i\right)=\frac{1}{m}\sum_{j=1}^{m}p\left(i,j\right).$$

When no marks are present along pixels with *i* coordinate, *L*(*i*) has its maximum value (i.e., all pixels are white); when marks are presents, *L*(*i*) has a lower value with 0 being the lower limit (i.e., all pixels are black). However, *L* is never white or black but, depending on the number of marks present on the paper, its value varies. Specifically, the more claw mark passages in a particular area the darker the paper will become. Consequently, *L*(*i*) represents the activity of the bird across the horizontal direction of the image. Since higher mark numbers correspond to lower *L* values, and given that *0 *≤* p*(*i, j*) ≤ 1, it is more convenient to define the intensity profile *I* as follows:

I(i)=1−L(i)∀i=1,…,n.

By definition, *I*(*i*) ranges between 0 (white) and 1 (black), with higher values of *I* corresponding to higher mark densities, and thus, higher activity recorded for the bird (Fig. 2C). Before using *I*(*i*) for orientation and activity evaluation, *i* was converted into degrees with *α*_*i*_ *= i × *360°/*n* and then smoothed with a 6° moving average filter to remove the potential artefacts due to presence of marks on the paper left during the paper handling (Fig. 2C). The filtered intensity profile was finally plotted as circular density plot (Fig. S3) and was thereafter used for circular statistics analysis (see below). The activity *A* was estimated as the mean of the intensity profile as follows:

$$A=\frac{1}{n}\sum_{i=1}^{n}I\left(i\right)$$

and reported as a percentage in circular plots (Fig. S3) or plotted for comparison with the other methods (e.g., Fig. S7).

### Evaluations by Video‐recording

#### Method 4. Video annotation

**Figure 3:**
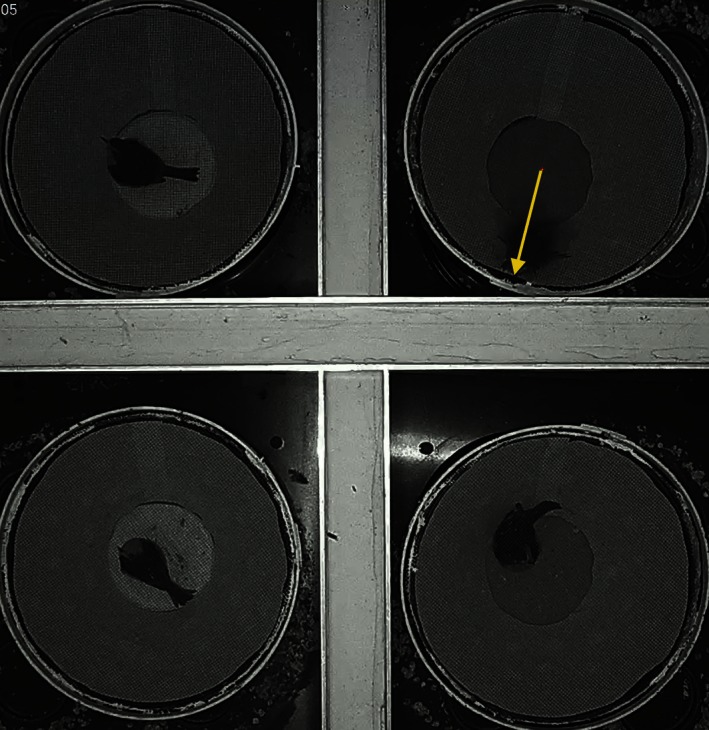
Screenshot of manual annotation procedure of the videos recorded during the orientation experiments performed in modified Emlen funnels (300 mm top diameter). The bird's orientation is annotated by manual clicking on the position of the beak of the bird during the attempt to take off (example shown for the top right cage).

The videos recorded during the experiment were imported in ImageJ software and manually annotated with the bird's jumps during the attempt of takeoff (jump and takeoff attempt are used with the same meaning through the text). Videos from each bird were annotated separately by visually defining the center of the cage and successively annotating the position of the bird's beak at the maximum distance from the cage center during the takeoff attempt. The beak position was represented as a vector with length equal to the distance of the beak from the cage center and the angle formed between the top of the image (0°) and the beak itself (Fig. 3). For statistical analysis, the lengths of the vectors were considered equal to 1 for all jumps, following methods outlined by Muheim et al. (2014). The annotated jump positions were represented as a circular scatter plot for individual tests (Fig. S4) and used for orientation evaluation. We estimated the activity as the sum of all the jumps recorded during the 1‐h assay (e.g., Fig. S7).

#### Method 5. Computer vision

**Figure 4:**
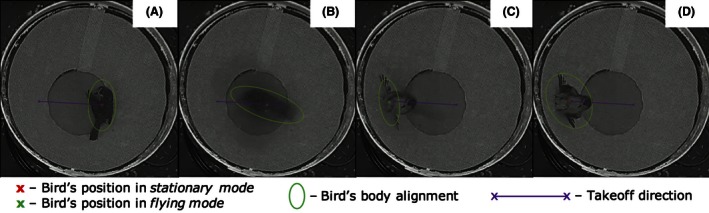
Four consecutive frames of a crop of a single cage of the video recorded during the orientation experiments showing features automatically extracted with the computer vision method. (A) Before taking off, the position of the bird is annotated as *stationary* mode (red cross) and the bird's body alignment is determined as the main axis of the ellipse fitted around its body. (B) The bird quickly moves during takeoff, and its position is tracked as *flying* mode (green cross). (C) The bird is still in *flying* mode while reaching the sloped wall of the funnel. (D) The bird hits the wall, and its position is now back to *stationary* mode (red cross). The takeoff direction is determined as the bird position before and after a mode transition that is from (A) to (D), and it is reported (purple line) on all four panels. Notice how the direction of takeoff is taken from the bird's position before taking off in (A) and not from the center of the cage as for the manual annotation procedure (Fig. 3). (see also the example in Movie S1).

To evaluate automatically the behavior of recorded birds, we used modern image processing algorithms combined with novel filtering procedures using MATLAB ver. R2014b. The position of the bird for each cage was determined using a video segmentation procedure based on dynamic background subtraction (for details see Appendix S1 and Felsberg et al. 2015). The bird's body alignment was determined as the main axis orientation of the ellipse fitted to the bird's body (Fig. 4).

Successively, the status of the bird was estimated as in either *stationary* mode or *flying* mode using a filter based on a jump Markov model as described in Gustafsson (2010) combining the bird position provided by the image processing with the additional indicators. In the *stationary* model little movement is assumed, which is modeled as low variance in position, and low wing fluttering, whereas in the *flying* mode, significant movement is assumed, which is modeled as high variance in position, and high wing fluttering. As the funnel guides the bird back to the center after the attempted takeoff, the bird's radial distance from the funnel center was also used in the model, assuming a position near the center for the *stationary* mode and near the outer perimeter for the *flying* mode. To account for the projection effect of the camera, the filter estimated the position of the bird in real‐world coordinates. To relate a real‐world coordinate (in mm) to the bird position in the image (in pixel), a perspective model was implemented using standard camera geometrical modeling procedures (Ma et al. 2012). Given the model and data, we used a filter bank of extended Kalman filters as described in Gustafsson (2010) to estimate the most likely sequence of modes. A formal description of the filtering procedure is available in Appendix S1.

Given the bird position before and after a mode transition, the direction of takeoff was determined as the angle between the top of the cage (0°) and the takeoff direction projected on the ground (Fig. 4). The takeoff directions for an individual test were represented as a circular scatter plot (Fig. S5) and used for orientation evaluation. The activity for the computer vision method was estimated as follows: (1) the total number of jumps recorded for each 1‐h assay, (2) the total distance moved (Muheim et al. 2014) and (3) the flying time expressed as a fraction for each assay (Figs. S7 and S10). The flying time was determined as the time spent in *flying* mode (see above) and uses variance of the pixel values in the difference image as a measurement of wing fluttering (see Appendix S1).

### Data analysis

Results were evaluated using R software version 3.1.1 (R Core Team, 2014). The mean orientation for each test was calculated using circular statistics according to Batschelet (1981) using the *circular* R package (Agostinelli and Lund 2013). The orientation data comparison between the five methods was examined with correlation analysis using the circular version of the Pearson's product‐moment correlation test (Jammalamadaka and Sarma 1988). We compared the level of activity measured by the five methods using the repeated‐measures ANOVA after the activity estimates were normalized within each method.

## Results

### Thermal paper evaluation

#### Manual counting

The difference in the angle of orientation estimated by the two persons (experienced and inexperienced) was in the range of 2–4° for all thermal paper sheets (Fig. S1), and the results were significantly correlated (*r* = 0.95, *n* = 12, *P* = 0.027). Although an apparent tendency of the naïve evaluator was to underestimate the birds’ activity level compared to the experienced user (Figs. S7 and S9), the repeated‐measures ANOVA of number of scratches counted for each bird was not significant between the users (*F*_1,20_ = 1.319, *P* = 0.264). These results indicate that, for our experimental design, the user experience did not affect the outcome of the results, neither for orientation nor for activity estimation. For this reason, and to avoid pseudoreplication, we will only use the results of the experienced user for comparison with the other methods in the following sections.

#### Visual estimation

**Figure 5:**
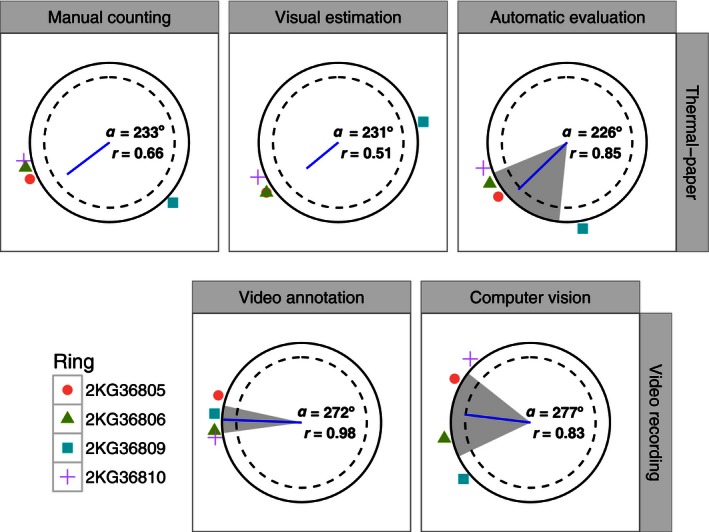
Mean orientation of European robins recorded in modified Emlen funnels obtained with five different methods. Each dot outside the unit circle indicates the mean orientation of a single bird for all three assays performed. The blue lines show group mean angle (*α*) drawn in the unit circle relatively to the mean vector length (**r**). The dashed circles indicate the minimum length of the mean vector needed for 5% significance according to the Rayleigh test (Batschelet 1981). The 95% confidence interval (gray area) is reported for significantly oriented distributions. Comparison between methods is presented as correlation table in Figure S8.

**Figure 6:**
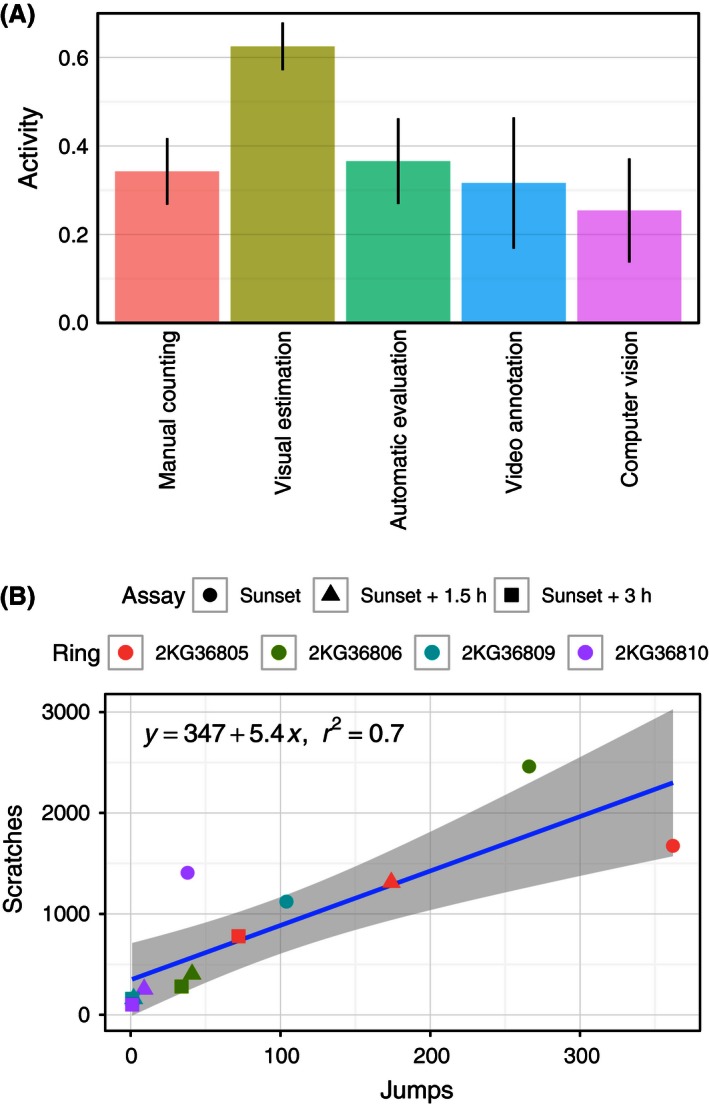
(A) Comparison of different methods to estimate the activity expressed by European robins in circular cages. Results are reported as birds’ mean ± SE (*n* = 4) of three consecutive 1‐h assays performed at sunset and after 1.5 h and 3 h. Activity values are normalized within each method for comparison purpose. Differences between methods are discussed in the text. (B) Relationship between number of scratches manually counted on thermal paper and number of jumps detected by computer vision algorithm for all birds and assays. Linear regression (line and equation) and 95% confidence interval (shaded area) are also reported.

The same experienced user evaluated the thermal paper sheets using both the more time‐consuming manual counting method and the faster visual estimation method. The comparison between the two methods showed a significant correlation in the orientation evaluation (Fig. S8), with almost identical estimation of the mean group angle (Fig. 5). However, the level of activity estimated by the two methods were different (*F*_1,20_ = 15.10, *P* < 0.001) with the visual estimation method (Mouritsen 1998) overestimating the average activity compared with the manual counting method (Fig. 6A).

#### Automatic evaluation

The automated image analysis of the thermal paper was in accordance with both the manual counting method and the visual estimation method (correlation shown in Fig. S8). The mean group orientation was in the southwest direction with only a few degrees of difference compared with the manual counting and the visual estimation methods (Fig. 5). The activity estimation based on the automatic evaluation was not different from the manual counting method (*F*_1,20_ = 0.074, *P* = 0.789), but significantly different from the visual estimation method (*F*_1,20_ = 10.05, *P* < 0.005) with the latter one overestimating the mean birds’ activity (Fig. 6A).

### Video‐recording evaluation

#### Video annotation

The orientation results of the manual annotation of video recorded during the experiment showed no correlation with any of the three methods used for evaluating the thermal paper (Fig. S8). Furthermore, the activity estimation, measured as the total bird's takeoff attempts, was not different from the manual counting and the automatic evaluation of thermal paper (*F*_1,20_ = 0.055, *P* = 0.817 and *F*_1,20_ = 0.174, *P* = 0.681, respectively). However, the measured activity differed from the visual estimation method (*F*_1,20_ = 8.239, *P* < 0.01; Fig. 6A).

#### Computer vision

As for the video annotation method, the computer vision results of the birds’ orientation were not correlated with any of the three thermal paper methods (Fig. S8). However, the results were in agreement with the video annotation method both in the visual inspection of the data distribution (Fig. S6) and in the correlation analysis (Fig. S8). Furthermore, the mean orientation for the group based on computer vision was almost coincident with the video annotation (Fig. 5).

Computer vision provided us with three different methods to estimate the activity of the birds: (1) total number of jumps (or takeoff attempts), (2) distance moved, and (3) flying time. However, the alternative methods were all equivalent to detect the level of activity for all birds (repeated‐measures ANOVA: *F*_2,31_ = 0.141, *P* = 0.870; Fig. S10). For this reason, we only compared the results in terms of number of jumps with the other four methods evaluated here (Fig. 6A). The activity measured with computer vision was not different from the manual counting (*F*_1,20_ = 0.906, *P* = 0.353), automatic evaluation (*F*_1,20_ = 1.241, *P* = 0.279) and video annotation methods (*F*_1,20_ = 0.254, *P* = 0.620) but resulted in lower activity as compared to the visual estimation method (*F*_1,20_ = 17.96, *P* < 0.001) of the thermal paper (Fig. 6A).

Using computer vision, we were also able to detect the body alignment of each bird (Fig. S5). In particular, the data distribution of body alignment (at the frame prior to the takeoff attempt) was analyzed for concentration parameters. Although the birds were significantly oriented in their attempts to takeoff, the body alignment was not different from random (Fig. S11).

#### Temporal resolution

**Figure 7:**
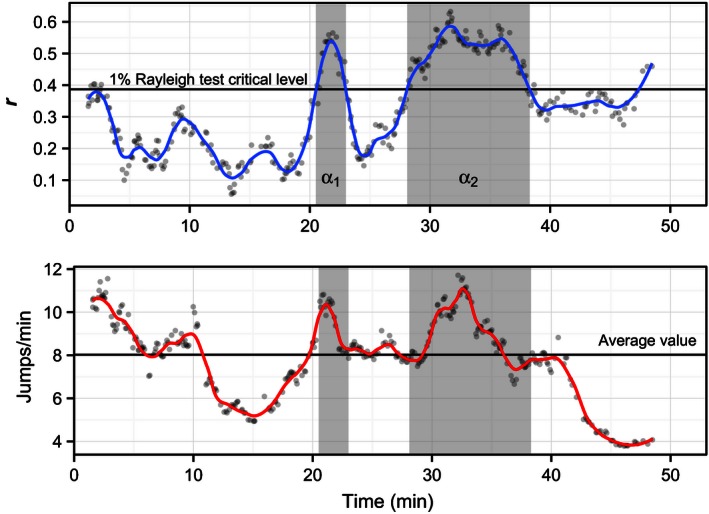
Example of temporal information obtained for one assay (Sunset, bird ring 2KG36805) using computer vision data. The mean orientation vector length (**r**) and the bird activity (expressed as jumps min^−1^) are calculated using a moving window of 30 jumps (gray points) and presented as continuous line using a local regression smoothing. In the upper panel, when **r** is above the critical level of the Rayleigh test (1% level reported as black horizontal line according to Batschelet 1981), the bird shows directionality (i.e., the mean direction is not random). The shaded areas indicated with *α*_1_ and *α*_2_ represents the time intervals when the bird shows orientation significantly different from random. In the lower panel, the mean jumps frequency is also reported (as a black horizontal line) to show higher and lower levels of activity.

Video analysis also provided as additional information the temporal resolution of both orientation and level of activity for each bird tested. An example for a single bird is reported in Figure 7. In this case, the bird is highly active in the first 10 min of the experiment during which time it is not showing any preferred direction. Later, in two different occasions, the bird showed high activity and significant directionality (Rayleigh test, *P* < 0.001) for several minutes (cf. shaded areas in Fig. 7). The mean direction for the two intervals were 11° (*n* = 23, **r** = 0.52) and 39° (*n* = 86, **r** = 0.49), respectively. Toward the end of the recording, the bird was again disoriented and showed only low activity in the cage (Fig. 7). The two intervals with high activity clearly influenced the result of the video analysis for this assay and bird (Sunset, ring number 2KG36805). In fact, with both video analysis methods, the mean orientation was in the northeast direction (Figs. S4 and S5). On the other hand, all methods using the thermal paper resulted in a mean southwest direction (Figs. S1–S3). This difference points at the fact that takeoff attempts are not always well correlated with the evaluation of scratches left on thermal paper (see also Fig. S8). This difference is, however, not true when comparing the activity‐level estimation with the different methods (Fig. 6A). Indeed, the activity level measured by counting the scratches on thermal paper is well correlated with the number of takeoff attempts detected by the computer vision analysis (Fig. 6B). However, it is worth noting that the range of number of scratches for a single jump was between 5 and 159.

## Discussion

### How does video analysis compare with thermal paper evaluation?

Although all the methods explored provided orientation results compatible with the expected migratory direction of the studied species, the European robin, in our experimental location (either southwest or west; Sandberg et al. 1988; Åkesson et al. 2015), the analysis of video recorded during the experiment and the evaluation of the thermal paper sheets were not correlated (Fig. S8). Moreover, only three methods resulted in mean orientations for single experiments being not different from random (Fig. 5).

We did not use the above‐mentioned results for drawing any conclusion because for a quantitative analysis a larger data set will be required. Furthermore, the two comparisons mentioned above do not imply that one method was better than the others, but simply points to the fact that each method registers the bird's orientation behavior differently. Indeed, the thermal paper recorded any activity of the bird while moving up on the sloping walls of the funnel‐shaped cage. By simply inspecting the video recorded (see example in Movie S1), we realized that once the bird has performed a takeoff attempt, it does not always stop flapping the wings when hitting the net on top of the cage, but more often keeps trying to fly for several seconds producing multiple scratches on the paper with its claws. Moreover, the scratches were produced not only in the takeoff direction, but as the cage is circular, the birds fluttered around leaving claw marks in multiple directions. On the other hand, the video analysis allowed us to detect the single takeoff attempt and precisely measure the direction the bird intended to fly (also shown in Movie S1). In addition, video analysis allowed for detailed temporal resolution information that provided a better understanding on how the bird was behaving and this information was very useful in presence of highly scattered data as in the example reported in Figure 7. Scattered data are often discarded before testing group orientation reducing the overall sampling size. Whereas, with videos analysis, activity and orientation can be evaluated simultaneously (cf. Fig. 7) and scatters in both parameters discarded before the analysis.

We could confirm that the thermal paper is recording not only the takeoff attempts, but also the overall activity of the bird in the specific funnel sectors. The aim of this study, however, was not to determine whether this is a relevant measure of the birds’ orientation ability, but rather point out the differences among methods in tracking the bird behavior including activity. Although, the number of scratches on thermal paper was correlated with the number of takeoff attempts recorded on video, we suspect that differences in orientation results were biased by the fact that for a single jump were usually counted several claw marks. Thus, claw marks were not independent from each other, whereas each jump was likely not influenced from the previous one. Indeed, after the jump, the bird was “pushed back” in the middle of the cage as they moved down across the sloping wall of the funnel in a different direction than its previous jump. Furthermore, before jumping, the bird rotates around its body axis before selecting a new takeoff direction. Both the above phenomenon are clearly visible in the Movie S1 and are further supported by the fact that the body alignment of the bird before jumping was not different from random (Fig. S11). Indeed, in our setup, body alignment of the bird and orientation measured in the Emlen funnel are different phenomena and thus cannot be used interchangeably as previously reported (Begall et al. 2013).

Furthermore, when the computer vision method was used, the activity of the birds was quantified not only in terms of number of jumps (or takeoff attempts), but also measuring the distance moved by the bird or the amount of time the bird was spending in flight that may provide a better understanding of the birds’ behavioral expressions during migratory activity (Zugunruhe) (Berthold 1996).

### Are the estimations of activity in Emlen funnels influenced by user experience?

We did not find any difference in the results of manual thermal paper counting between the experienced user and the naïve user, both in the orientation and in the activity estimation. This was comforting as counting claw marks is time‐consuming and will allow for multiple users performing counting to speed up data evaluation. It should be stressed, however, that in our case the experienced user directly instructed the naïve user and both used the same procedure and annotation sheet with 24 sectors. Thus, we cannot exclude that evaluations performed in different laboratories may result in differences due to dissimilarities in materials or procedures used.

### How well is the visual estimation method in describing the orientation of circular distributions?

The evaluation of orientation using the visual estimation of thermal paper as outlined by Mouritsen (1998) was well in agreement with the manual counting in 24 sectors (Åkesson et al. 1995) and the automatic evaluation of thermal paper using image analysis outlined in this study. Moreover, it was also the fastest manual method tested and needed only few minutes for each thermal paper sheet for the final evaluation. The only shortcoming with this method was the overestimation of activity compared with all other methods (Fig. 6A). The overestimation of activity was predominantly a result of the limited resolution with only four levels of activity used in this method, which resulted in us overestimating activity for birds showing low levels of activity (cf. Fig. S12). The visual estimation method, thus, needs to be used with caution when both activity levels and concentration parameters are used in the statistical analysis.

### Is image analysis a more convenient method to analyze thermal paper?

The automatic evaluation of thermal paper based on image analysis was very efficient and provided high angular resolution. For a single digitalized paper sheet, the computation time was of the order of milliseconds and the resolution was about 1/12° at 600 dpi scanning resolution. However, we only had available a flat scanner that was smaller than the thermal paper and consequently each paper sheet was scanned twice, one half‐side per time. For the future, the procedure can be shortened by simply using a larger flat scanner to skip the double scanning and stitching procedure.

A further improvement for this method would be to have an automated selection of the circular shape of the thermal paper. Although not very time‐consuming, having this last part of the procedure automated would make the entire method user‐free. This means that such a method would not only provide the fastest way in evaluating the thermal papers used during orientation experiments with Emlen funnels, but will be completely user‐independent making it possible to compare results from different studies.

This method represents an easy step forward to upgrade the current Emlen funnel studies using common digital image acquisition (i.e., office scanner) and freely available automatic image analysis algorithms (Kocsis et al. 1991; Abràmoff et al. 2004, Preibisch et al. 2009).

### Can manual video annotation replace the use of thermal paper?

The video annotation method was the most demanding method in terms of time invested to track the birds’ orientation and activity. To have unbiased results, a single user annotated each recorded bird for a total of 12 h of videos. The entire procedure took several days because, when birds were very active, it was not easy to detect the transition from stationary to flying state and the video had to mainly be inspected in slow motion. This method, thus, must be limited in study with a small sampling size. However, in our study, it was very important to include the manual video annotation to validate our computer vision algorithm.

### Are automated video‐tracking algorithms ready to take over the traditional paper method?

Computer vision is nowadays applied in many areas of animal movement research (e.g., Dell et al. 2014, and reference therein). In our case, an extremely simplified algorithm could have been based on extracting all positions of the bird when fluttering on the sloped walls of the funnel and use the measurements to compute an average takeoff direction. This approach would likely provide similar, if not identical, results to the traditional thermal paper analysis. However, we realized that such information is quite biased due to the limiting space the birds has to manoeuver.

A method to detect the single takeoffs could be the state machine approach where, setting up deterministic rules, it would be possible to determine the transition from stationary to flying state of the bird based on its position within the cage (e.g., Muheim et al. 2014). Relying only on the bird position, however, does not make such an approach robust and depending on image quality and bird behavior, it is sometimes difficult to capture the state transition. However, adding more indicators, such as wing flutter and other movement measurements makes the state machine approach complicated and heuristic. On the other hand, our method is based on modeling the different behaviors of the bird, incorporating several information sources, and applying modern filtering techniques to estimate the status of the bird. Such a method required a joint collaborative effort between biologists and experts in sensor data analysis. We provided, in Appendix S1, all the details to implement this multidisciplinary approach in other laboratories. However, we believe that with an increasing demand from the research community of such technology, new software will be designed also to be user‐friendly to nonexperts and computer vision will be included as routine procedure for orientation analysis as is currently happening for many behavioral assays (see examples in Dell et al. 2014; cf. Muheim et al. 2014).

Computer vision is a continuously growing discipline and newly developed software will become more easily available (Schneider et al. 2012; Dell et al. 2014; Muheim et al. 2014). Indeed, video‐recording of experiments will allow for successive reanalysis of the data as new algorithms will be implemented. One of the possible future additions to our method outlined here may be the tracking of the bird's head. Head scanning behavior has been shown to be a relevant behavior of the geomagnetic field detection in songbirds (Mouritsen et al. 2004); however, it has only been studied as a separate phenomenon and not combined with recording of orientation and activity data. We believe that the great potential of computer vision is the ability to detect and track more exhaustively the complexity of the behavior of captive birds without limiting the studies to only a few aspects, such as orientation (Muheim et al. 2014) or head scanning (Mouritsen et al. 2004).

Ultimately, computer vision does not require the use of thermal paper allowing orientation assays to not be limited to, for example, one‐hour duration and thus performed under the assumption that the time interval chosen for the assay is the one when the tested individuals are migratory active. Indeed, with video analysis, it will be possible to evaluate afterward when and if the bird showed any orientation during the duration of the experiment (e.g., Fig. 7). Furthermore, a setup not based on thermal paper can make use of larger circular cages and thus allow the tested birds to move more naturally with enough space for manoeuvring during the takeoff attempt possibly reducing the scatter in the data. Larger cages can also be equipped with food and water dispensers (e.g., Ilieva et al. 2016) and may be equipped for long‐lasting experiments allowing to combine studies of ecophysiology and migratory behavior.

### Comments and recommendations

We showed that the five tested methods record the songbirds’ orientation behavior differently, in particular when comparing the thermal paper and video‐recording methods. With any method tested, the orientation was estimated quantifying a parameter (e.g., number of scratches or number of jumps) that is assumed to be related to the orientation choice of the bird. In our study, we found support that the number of jumps were likely independent from each other and not (or less) affected by pseudoreplication. Thus, the video‐recording method should provide a more reliable estimation of the mean orientation for our tested birds. We chose as target species the European robin (Fig. 1) because it is historically the most used species in orientation experiments. However, a single species cannot provide the entire range of differences among the five tested methods. For example, species like the Willow warbler (*Phylloscopus trochilus*) flutters a lot, which may result in underestimation of their activity using the thermal paper, even with the automatic evaluation method. However, in this circumstance, the video analysis may be useful to disentangle the fluttering (using either the distance moved or the flying time) from the selection of the takeoff direction. Indeed, for our robins, distance moved, flying time and number of jumps provided with the same activity estimations. However, with species like some warblers, this may not be the case. Thus, our suggestion is to consider video analysis as complementary source of information when it is used with different species or different settings and use a subset of the data to select which parameter to use in the successive analysis of the entire dataset. We encourage to include video‐recording in future studies using the Emlen funnel and to scan the thermal paper after it has been manually evaluated. Such an approach would provide valuable data that can be analyzed to compare methods quantitatively and with a larger variety of species.

## Conflict of Interest

None declared.

## Supporting information


**Figure S1.** Results of manual counting of marks left by claws on thermal paper.
**Figure S2.** Results of visual estimation of marks left by claws on thermal paper.
**Figure S3.** Results of automatic estimation of marks left by claws on thermal paper.
**Figure S4.** Results of manual annotation of video‐recordings of bird activity during the experiments.
**Figure S5.** Results of computer vision analysis of the video recorded during the orientation experiments.
**Figure S6.** Comparison of the two methods used for analysis of videos recorded during the orientation experiments.
**Figure S7.** Results of different methods for estimating birds’ activity.
**Figure S8.** Correlation table of measured angles of orientation for the five methods evaluated.
**Figure S9.** Comparison of activity estimation of manual counting of different users (experienced vs. naïve).
**Figure S10.** Comparison of different methods to estimate the birds’ activity using computer vision.
**Figure S11.** Orientation and body alignment obtained with computer vision analysis.
**Figure S12.** Comparison of the five tested methods to estimate a single bird's activity.

**Appendix S1.** Image Processing and Filtering procedures.

**Movie S1.** Automatic analysis of an Emlen funnel orientation experiment.
