# Supplementary material for: Emlen funnel experiments revisited: methods update for studying compass orientation in songbirds
Source: Ecol Evol. 2016 Sep 7;6(19):6930–42. doi: 10.1002/ece3.2383 (PMC5513225; doi:10.1002/ece3.2383)
Supplement: Supplementary file 1 — Figure S1. Results of manual counting of marks left by claws on thermal paper. Figure S2. Results of visual estimation of marks left by claws on thermal paper. Figure S3. Results of automatic estimation of marks left by claws on thermal paper. Figure S4. Results of manual annotation of video‐recordings of bird activity during the experiments. Figure S5. Results of computer vision analysis of the video recorded during the orientation experiments. Figure S6. Comparison of the two methods used for analysis of videos recorded during the orientation experiments. Figure S7. Results of different methods for estimating birds’ activity. Figure S8. Correlation table of measured angles of orientation for the five methods evaluated. Figure S9. Comparison of activity estimation of manual counting of different users (experienced vs. naïve). Figure S10. Comparison of different methods to estimate the birds’ activity using computer vision. Figure S11. Orientation and body alignment obtained with computer vision analysis. Figure S12. Comparison of the five tested methods to estimate a single bird's activity. [file ECE3-6-6930-s001.pdf]

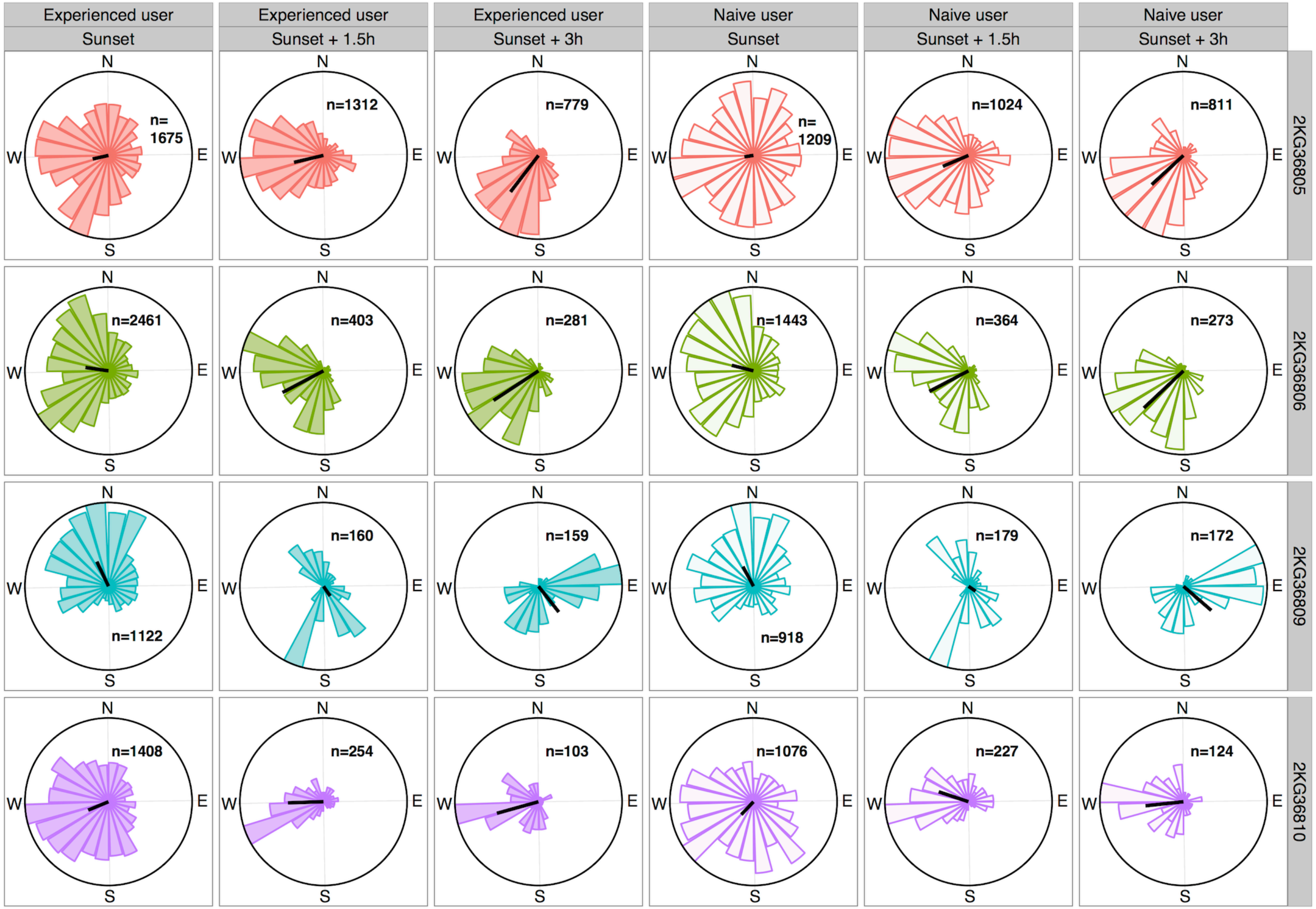

Figure S1. Results of manual counting of claw marks left on thermal-paper during the European robin orientation experiments performed in modified Emlen-funnels. Four birds (denoted by ring number) were used in three consecutive 1-hour assays performed at sunset and after 1.5h and 3h. Both an experienced and a naïve user (without previous experience in bird orientation procedures) independently counted the claws marks of each paper. Number of marks is reported as normalised circular histogram binned in 15° intervals used in the counting procedure (see text for details). Number of scratches (n) is reported for each plot. The black line indicates the mean distribution angle (α) and is drawn relatively to the mean vector length (**r**) varying between 0 and 1, with the size of the circle being equal to 1.

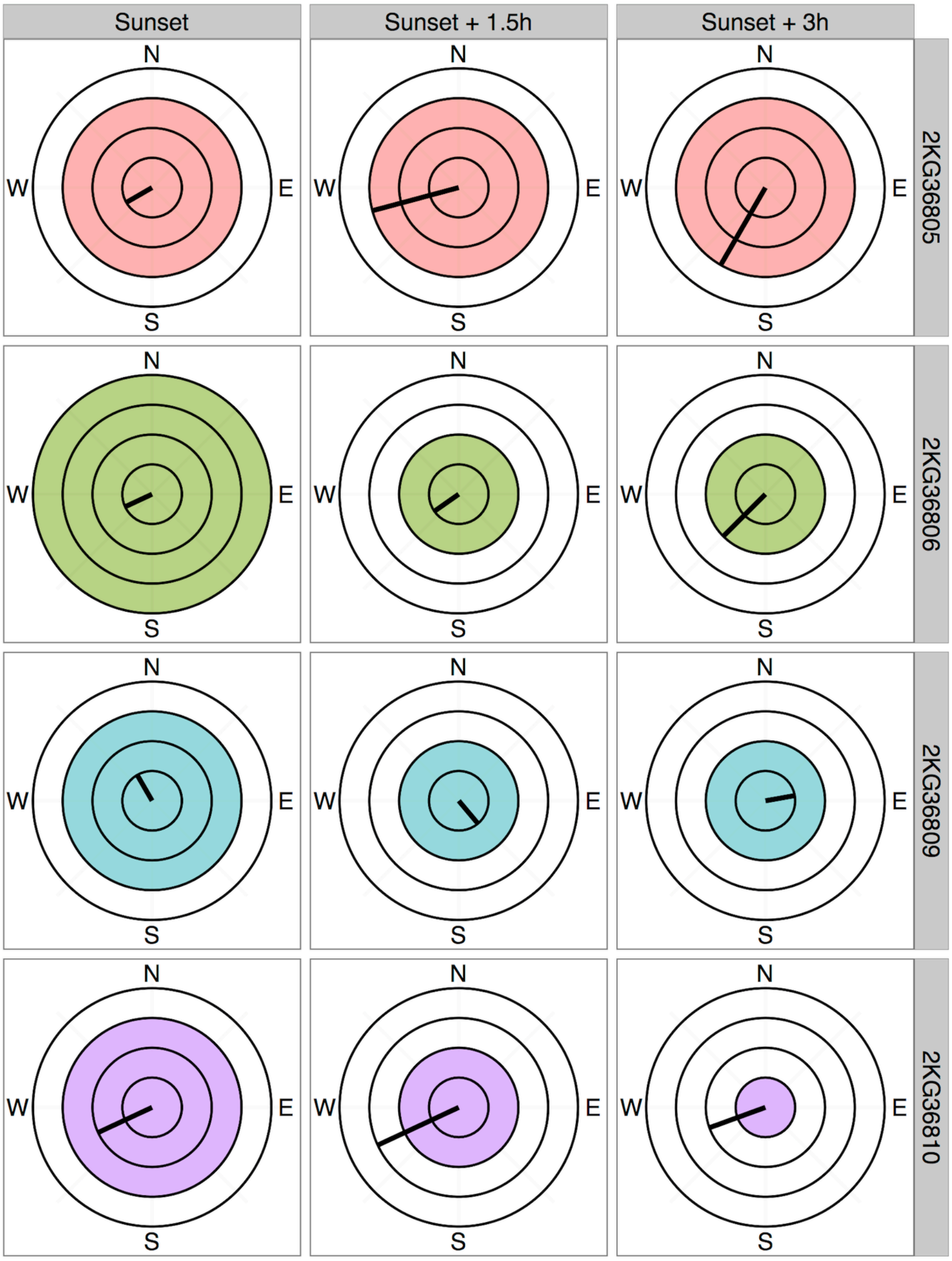

Figure S2. Results of visual estimation of claw marks left on thermal-paper during the European robin orientation experiments performed in modified Emlen-funnels. Four birds (denoted by ring number) were used in three consecutive 1-hour assays performed at sunset, after 1.5h and 3h, respectively. Results are reported as activity index (coloured shaded area) and concentration index (black line length). Both indexes are defined on a 0—4 scale (see text for description of procedure) represented by concentric circles. The black line is pointing to the estimated angle (α).

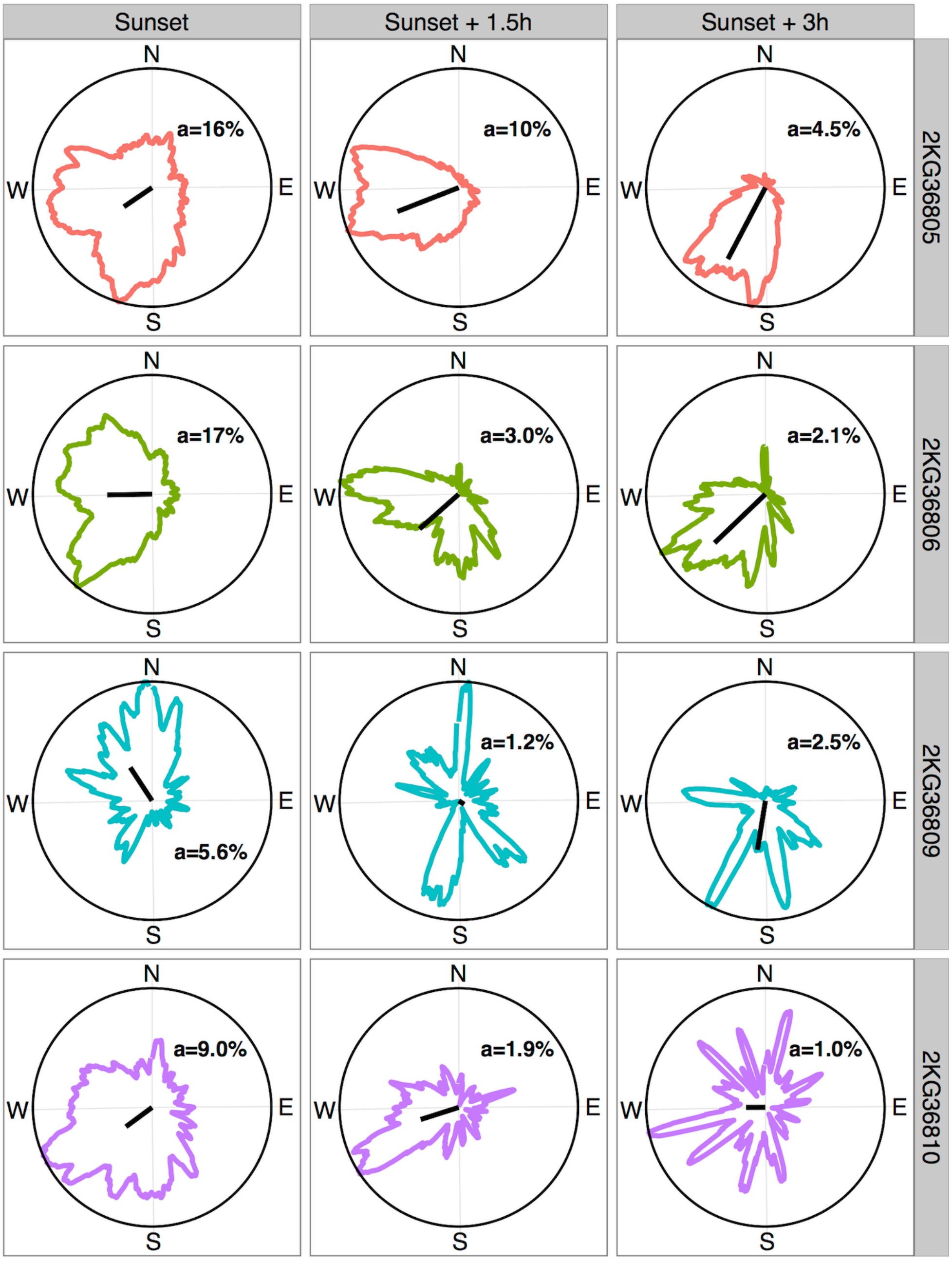

Figure S3. Results of automatic evaluation of claws marks left on thermal-paper during the European robin orientation experiments performed in modified Emlen-funnels. Four birds (denoted by ring number) were used in three consecutive 1-hour assays performed at sunset and after 1.5h and 3h. Angle of orientation is reported as normalised density plot of the intensity profile (see text for definition) of the digitalised scan of the thermal-paper. Level of activity (a) is calculated as mean intensity profile and reported for each plot as percentage (100% represents complete black paper). The black line indicates the mean distribution angle (α) and is drawn relatively to the mean vector length (**r**) varying between 0 and 1, with the size of the circle being equal to 1.

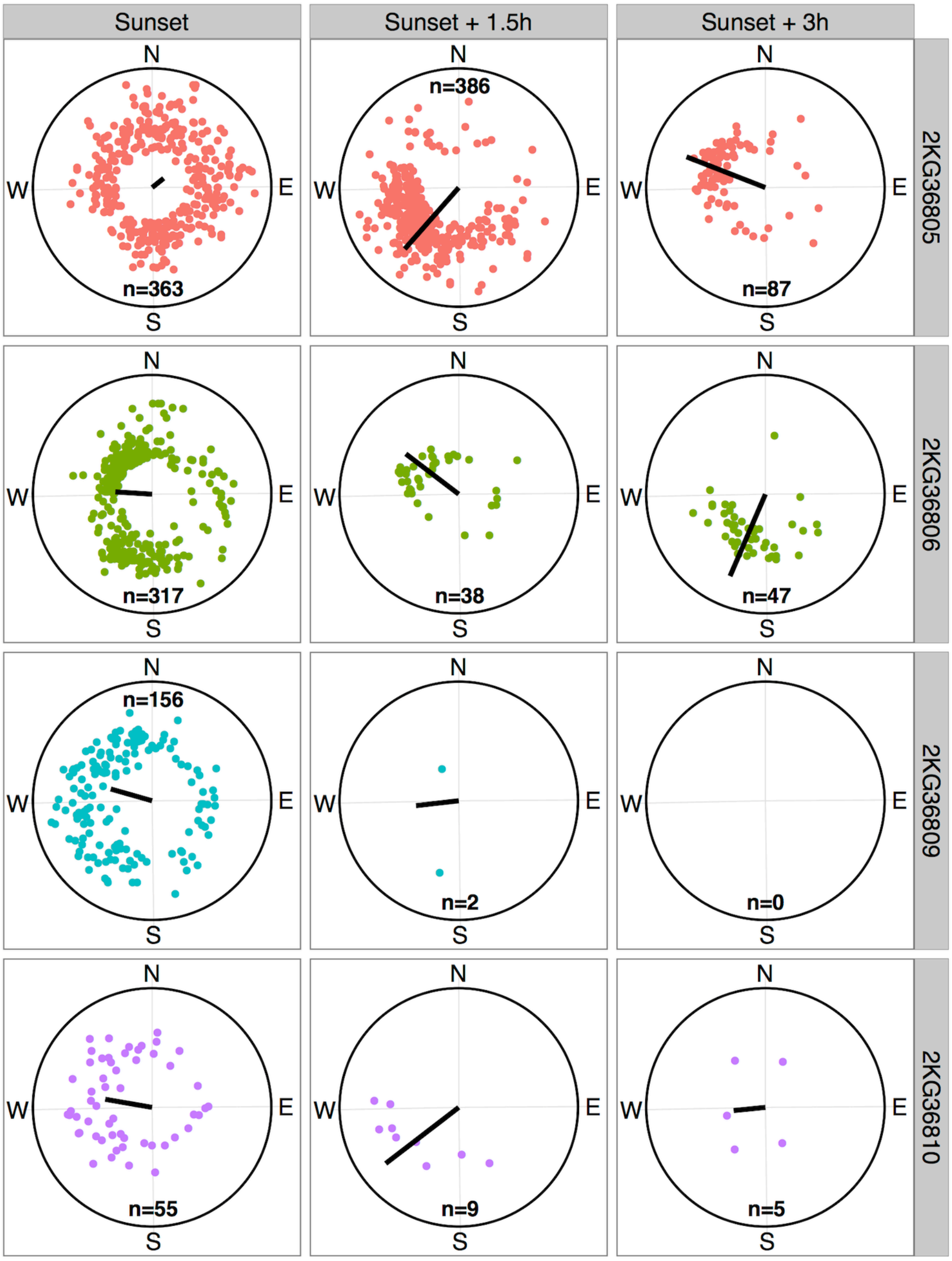

Figure S4. Results of manual annotation of the videos recorded during the European robin orientation experiments performed in modified Emlen-funnels. Four birds (denoted by ring number) were used in three consecutive 1-hour assays performed at sunset, after 1.5h and 3h, respectively. The positions of the beak of the bird during the attempt to take-off are reported as circular scatter plots with the circle being the diameter of the funnel-shaped cage (300 mm). Number of take-off attempts (n) is reported for each plot. The black line indicates the mean angle (α) and is drawn relatively to the mean vector length (**r**) varying between 0 and 1, with the size of the circle being equal to 1.

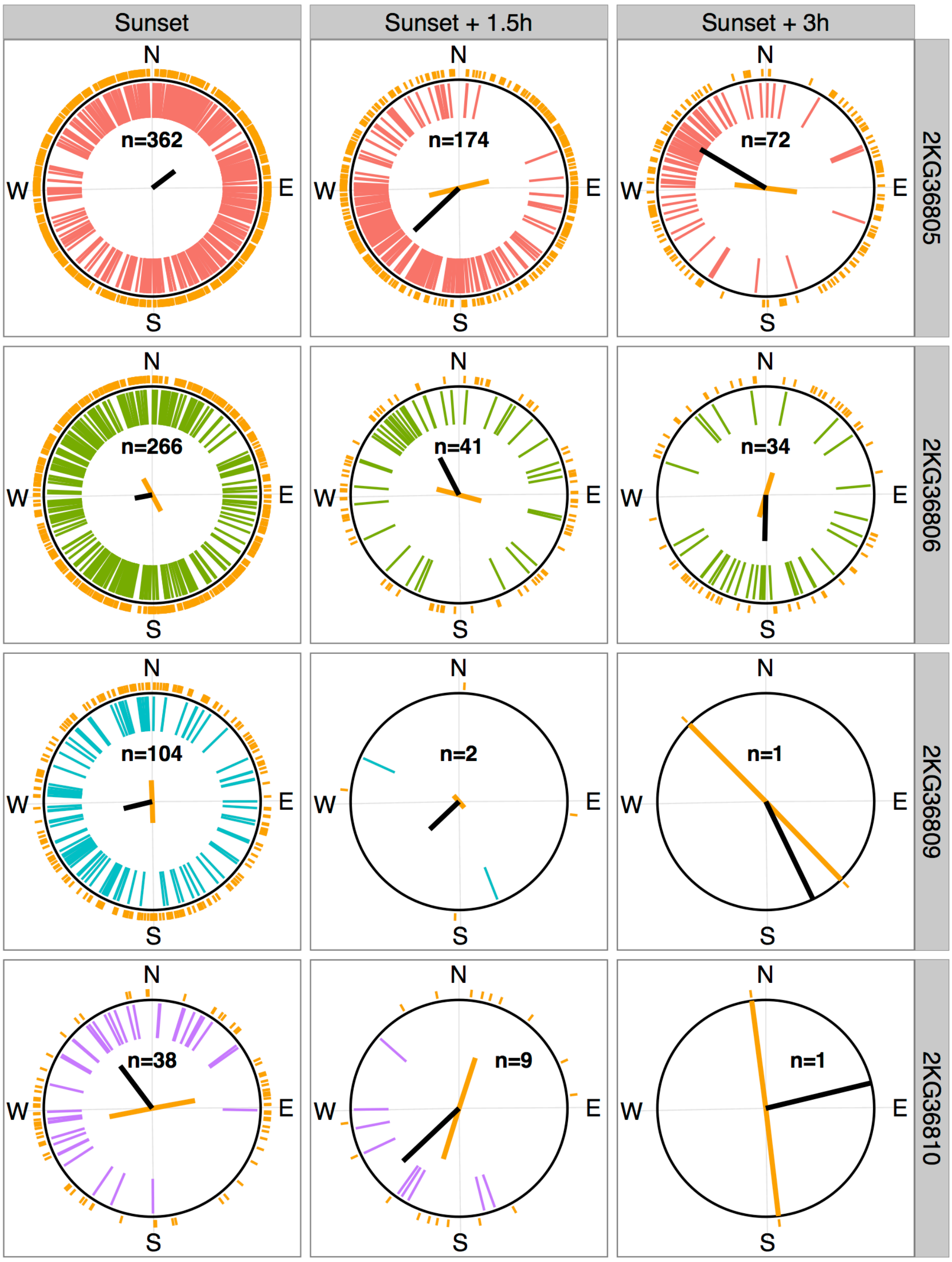

Figure S5. Results of computer vision analysis of the videos recorded during the European robin orientation experiments performed in modified Emlen-funnels. Four birds (denoted by ring number) were used in three consecutive 1-hour assays performed at sunset, after 1.5h and 3h, respectively. Take-off directions are reported as segments forming the angle between the top of the image and the take-off direction projected on the ground (see text for details) inside the circle. Bird's body alignment before each take-off attempt is reported as pair of short-lines located on opposite sites outside the circle. The black line indicates the mean take-off angle (α) and the orange line indicates the axial mean vector of body alignment. Black and orange lines are drawn relatively to the mean vector length (**r**) with the size of the circle being equal to 1. Number of take-off attempts (n) is reported for each plot.

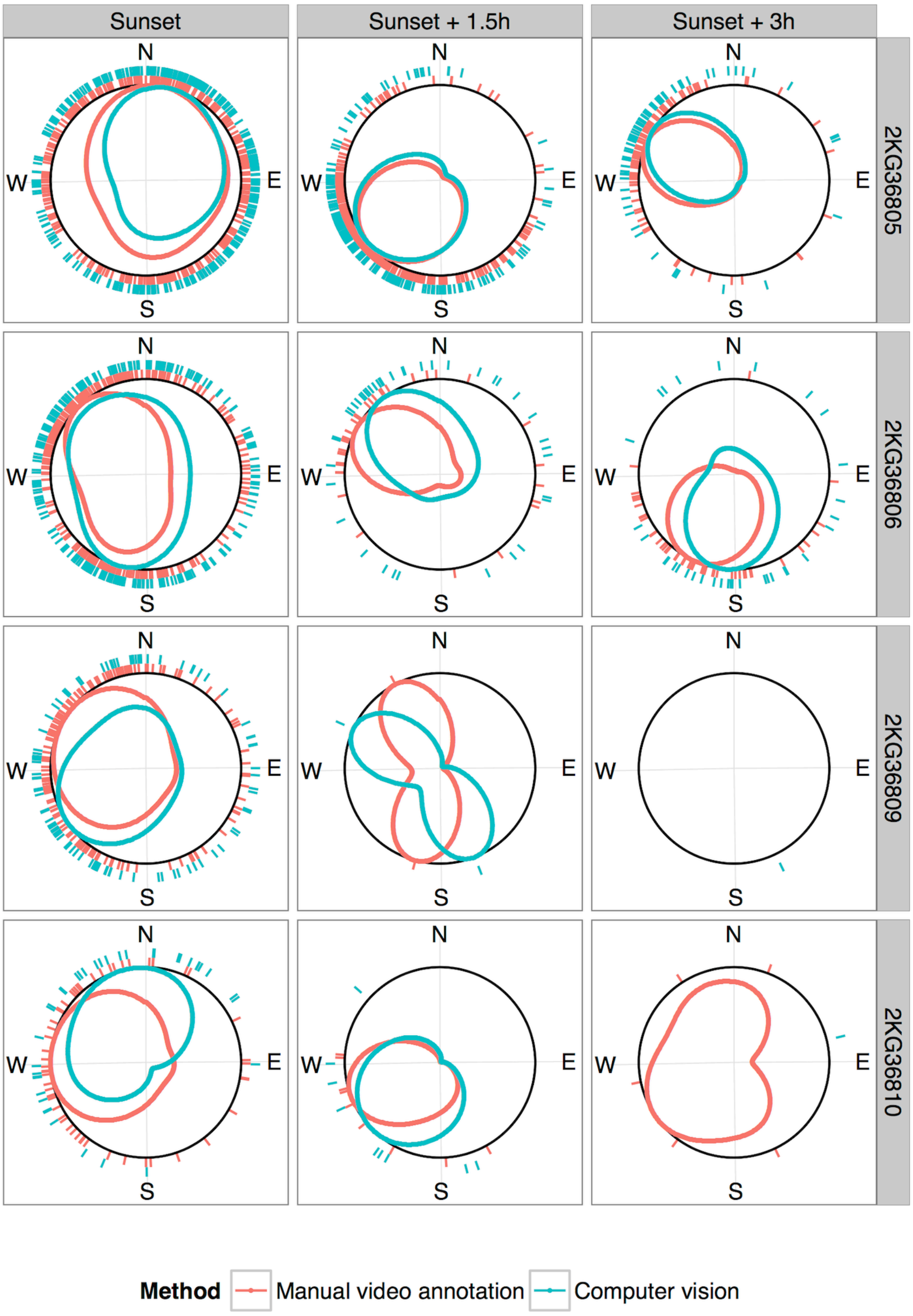

Figure S6. Comparison of the two methods used for analysis of videos recorded during the European robin orientation experiments performed in modified Emlen-funnels. Four birds (denoted by ring number) were used in three consecutive 1-hour assays performed at sunset, after 1.5h and 3h, respectively. Results are reported as normalised continuous angle distribution using a smoothing kernel around each observation. Observations for both methods are also reported outside the circle.

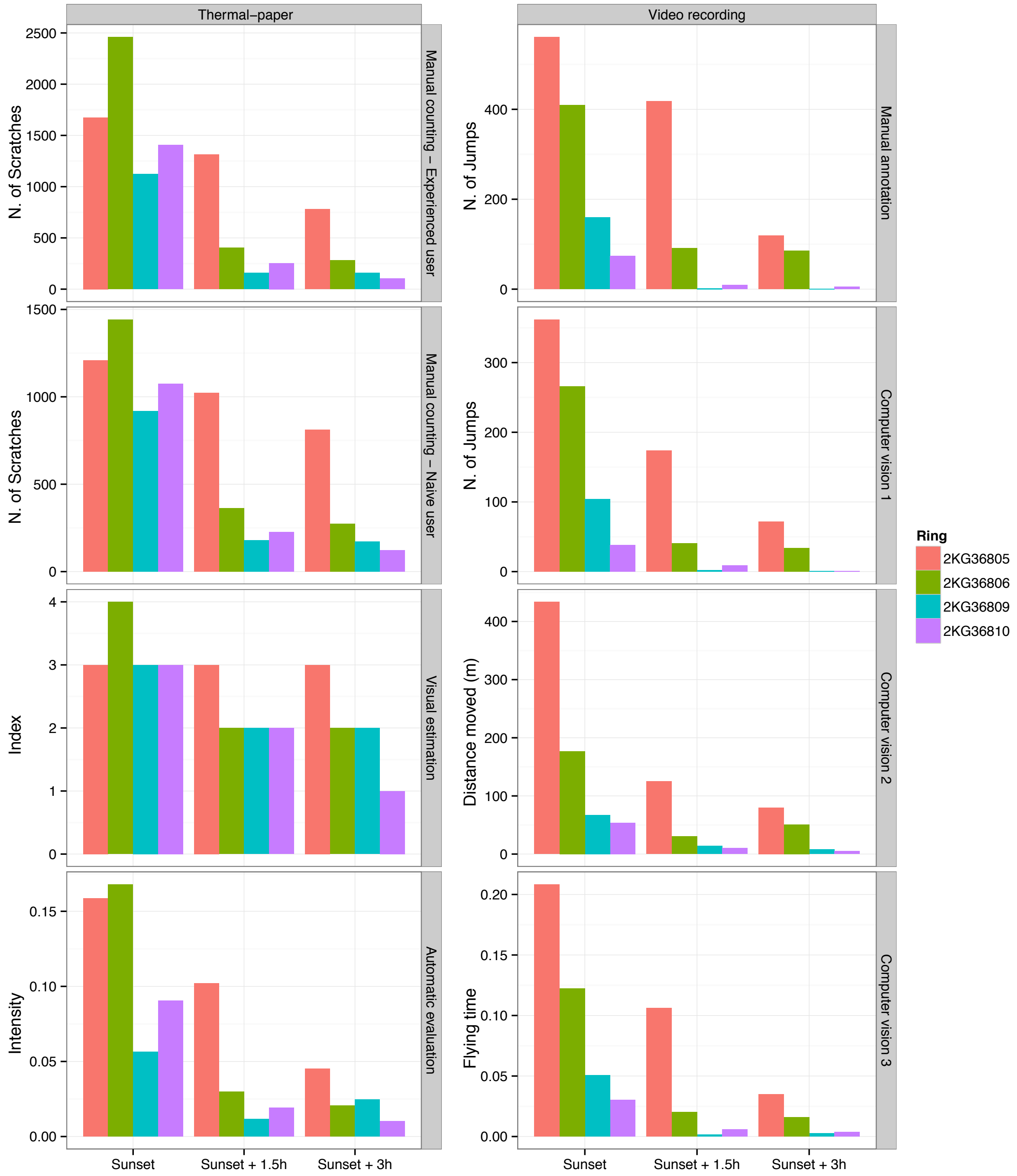

Figure S7. Results of different methods used for estimating birds' activity during orientation experiments performed with European robin in modified Emlen-funnels. Four birds (denoted by ring number) were used in three consecutive 1-hour assays performed at sunset, after 1.5h and 3h, respectively. Activity was estimated from the thermal-paper (left side) and video recording analysis (right side) with different methods (see text for details). Each method estimates activity in different units (see Y-axis); for comparison between methods see normalised values in Fig. S10 and Fig. S12.

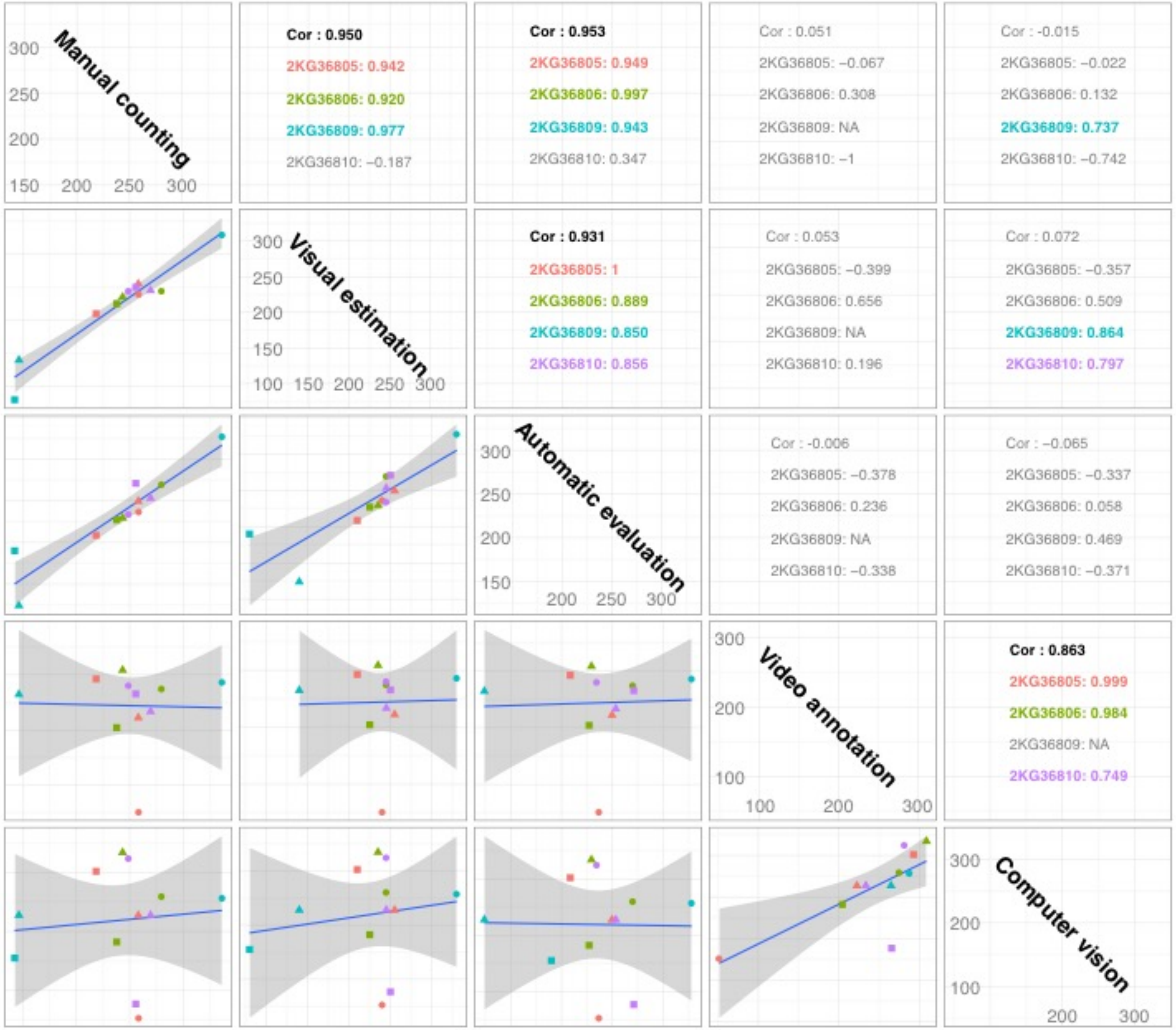

Figure S8. Correlation table of measured bird's orientation angles of the five tested methods. In the bottom-left is reported the scatter plots (unit in degree in both X- and Y-axis) of the four birds and three assays performed. The linear regression (blue line) and 95% confidence interval (shaded area) are also reported. The upper-right represents the correspondent coefficients of the Pearson's circular product moment correlation for all methods combinations. Significant positive correlations ($p < 0.05$) are reported in bold font for single birds (indicated by ring number, in colour, n=3) and for the entire dataset (in black, n=12).

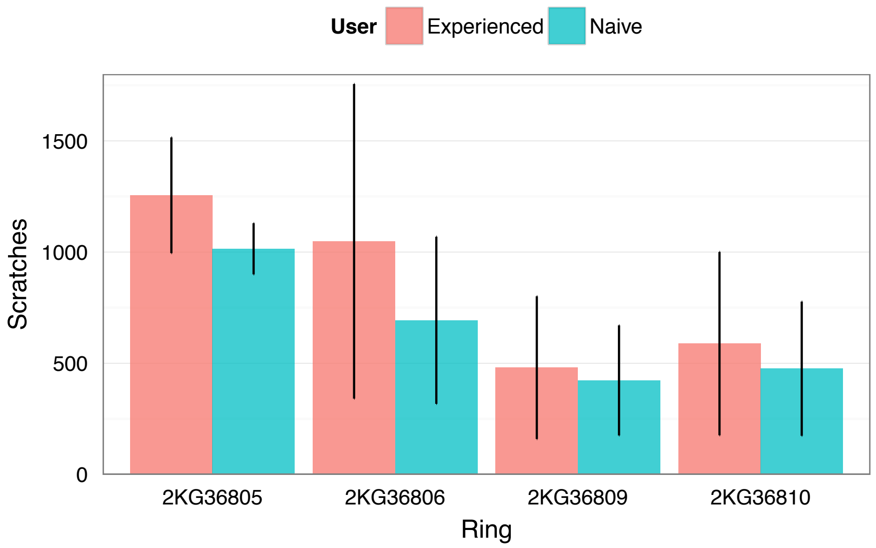

Figure S9. Comparison of activity estimation of manual counting of scratches left by bird's claws on thermal-paper. Results are reported for each bird (indicated by ring number) as mean ± SE of three consecutive 1-hour assays for both an experienced and a naïve user (i.e., without previous experience in bird orientation procedures).

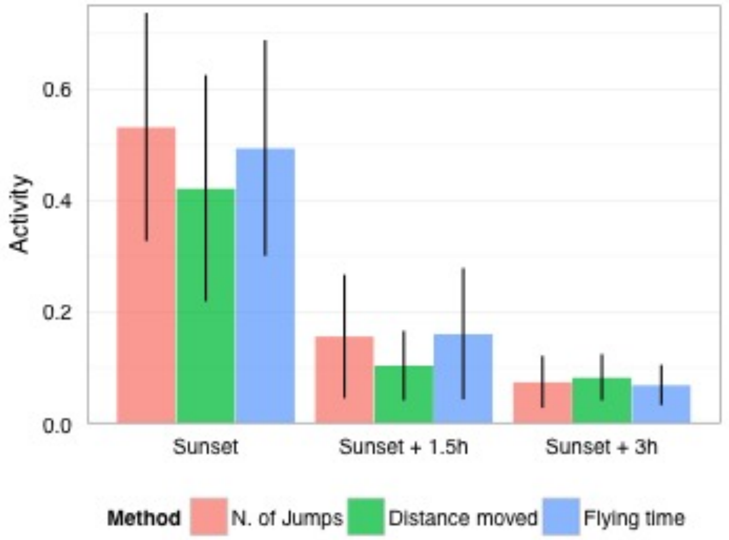

Figure S10. Comparison of different methods to estimate the birds' activity using computer vision. Results are reported for each bird (indicated by ring number) as mean ± SE of three consecutive 1-hour assays. Activity values are normalised within each method for the purpose of comparison.

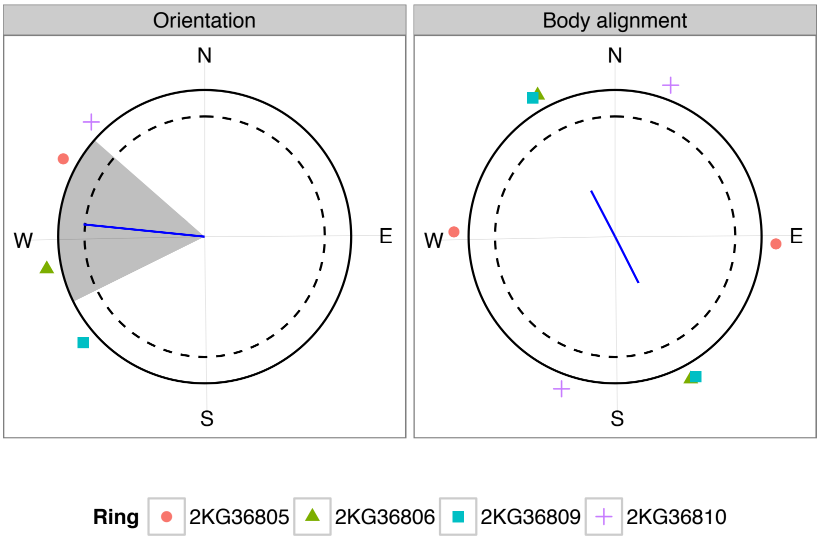

Figure S11. Orientation and body alignment obtained with computer vision analysis of video recorded during the experiment. Dots outside the unit circle indicate the mean angle of a single bird for orientation data and axial data (dots located in opposite direction) for body alignment. The arrows show group mean angle (α) drawn in the unit circle relative to the mean vector length (**r**). The dashed circles indicate the minimum length of the mean vector needed for 5% significance according to the Rayleigh test. The 95% confidence interval (grey area) is reported for orientation mean angle.

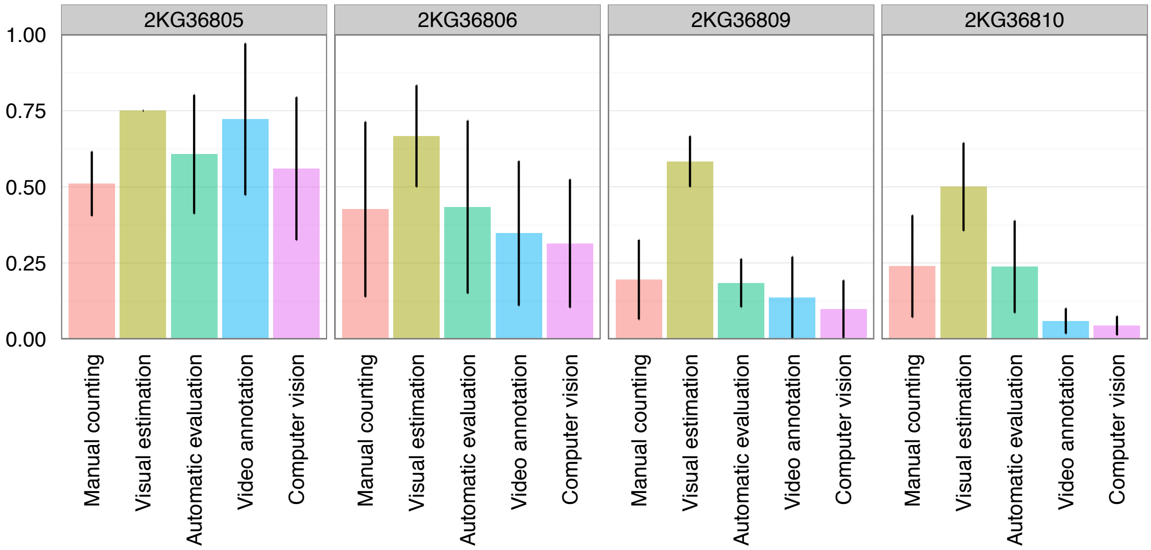

Figure S12. Comparison of the five methods used to estimate the birds' activity in circular cages. Results are reported in different panels for each bird (indicated by ring number) as mean ± SE (bar plot and black line, respectively) of three consecutive 1-hour assays. Activity values are normalised within each method for the purpose of comparison.
