# Supplementary material for: Emlen funnel experiments revisited: methods update for studying compass orientation in songbirds
Source: Ecol Evol. 2016 Sep 7;6(19):6930–42. doi: 10.1002/ece3.2383 (PMC5513225; doi:10.1002/ece3.2383)
Supplement: Supplementary file 2 — Appendix S1. Image Processing and Filtering procedures. [file ECE3-6-6930-s002.pdf]

# Appendix S1

This appendix describes the specific image processing and filtering algorithms used and references are provided for a more detailed description of the general algorithms.

## 1 Image Processing

The general approach is to first apply image processing sequentially for each frame in the video to obtain a segmentation of the bird in a manner that is robust to changing light conditions. This is achieved by dynamically estimating a model of the background for each pixel and then subtracting the background from the pixels to obtain an estimate of the foreground containing the bird. The bird position and extension is then extracted from the difference image as well as an estimate of the blurriness of the bird. This section outlines the technical details of the chosen method, however, other possible methods could be applied that would achieve similar results.

A moving quantile background model, see Buragohain and Suri (2009) for a general description, set at 90% intensity has been used for each individual pixel in the image sequence as lighting conditions change over the sequences. The update rate has been set low enough for the birds not to become a part of the background even during long periods of inactivity. The moving quantile background model keeps the background model value close to the set quantile when considering the distribution of pixel values over time. The quantile has been set to 90% as the background tends to have a higher intensity than the bird.

For each frame, the pixel-wise difference image is calculated, see Fig. A1. The difference image is summed over basis functions in a log-polar grid, see Fig. A2 and A3, similar to bins of a soft histogram. From the resulting coefficients, the peak of the strongest mode is obtained by channel decoding as in Forssén (2004). This mode becomes the estimated bird position, with a notably higher precision than the bin-spacing of the basis functions, see Felsberg et al. (2006, 2015). This process reduces influence of noise and outliers in other parts of the image.

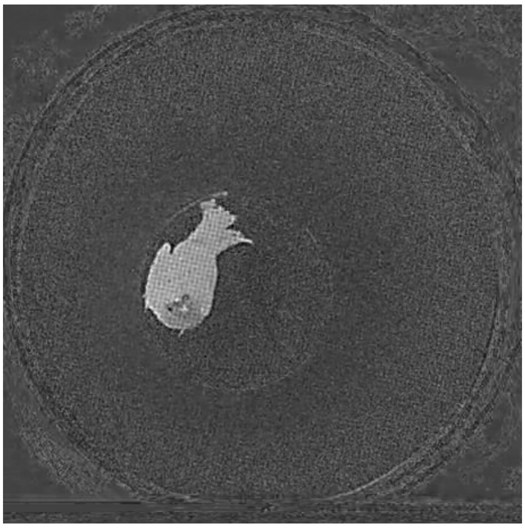

Figure A1: Example of a difference image, where the estimated background model has been subtracted from the frame.

The body orientation is determined by first calculating the 95% quantile of the difference image. Bird pixels are found as difference pixels deviating more than the 95% quantile threshold. An ellipse is fitted to the largest connected area of the bird pixels; its main axis orientation provides an estimate of body orientation. Additionally, the variance of the distribution of bird pixel values in the difference image provides an indication of wing fluttering. When the bird is active, motion blur of primarily the wings generates a wide distribution of deviation of pixel values. When the bird is stationary, the variance is small.

# 2 Filtering

Given the measurements from image processing, the next step is to determine when takeoffs occur and whether the bird is in *stationary* or *flying* mode. This can be estimated in a deterministic manner by setting up rules for the movement of the bird to indicate takeoffs, however, such methods tend to get complicated when several measurements of different types are available. The approach chosen is therefore to model the behaviour and measurements, such as position, amount of movement and blurriness, for each mode and apply a filter to determine the most likely mode over time. This allows for a soft combination of many different types of measurements, rather than making a hard decision. A transition from *stationary* to *flying* mode is then

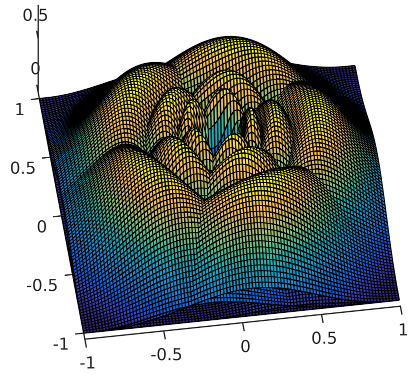


Figure A2: Example of basis functions using three radial and five angular bins (channels).

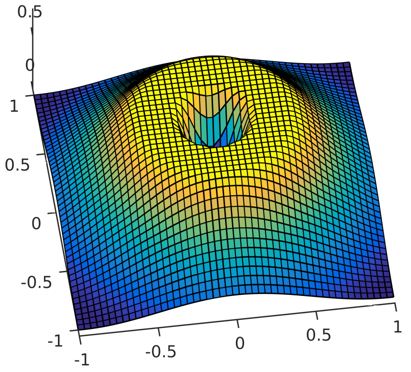


Figure A3: Sum of all basis functions, generating a smooth surface on a disc.

considered to be a takeoff. This section outlines the technical details of the chosen method, however, other possible methods could be applied that would achieve similar results.

The bird motion is modelled as a jump Markov linear model with the states and modes

$$\mathbf{x}_t = \begin{bmatrix} x_t \\ y_t \\ b_t^s \\ b_t^f \end{bmatrix} \quad \text{and} \quad \delta_t = \{s, f\}, \tag{1}$$

where the subscript $t$ denotes a discrete time corresponding to the video frame index, $x_t$ and $y_t$ represent the bird position in real world coordinates, $b_t^s$ and $b_t^f$ represent the wing flutter levels for the two modes, $\delta_t$ is the mode and $s$ and $f$ represent the *stationary* and *flying* mode, respectively. Note that the wing flutter levels are measured as the amount of motion blur and dependends on the lighting conditions in the video, which means the levels

change over time. The model is then defined as

$$
\begin{aligned}
\mathbf{x}_{t+1} &= \mathbf{x}_t + \mathbf{v}_t(\delta_t), \\
\mathbf{y}_t &= \mathbf{h}^{\delta_t}(\mathbf{x}_t) + \mathbf{e}_t(\delta_t), \\
\mathbf{h}^{\delta_t}(\mathbf{x}_t) &= \begin{bmatrix} \mathbf{h}_c(x_t, y_t) \\ b_t^{\delta_t} \end{bmatrix}, \\
z_t &= r(\delta_t), \\
\mathbf{v}_t(\delta) &\sim \mathcal{N}(0, \mathbf{Q}_\delta), \\
\mathbf{e}_t &\sim \mathcal{N}(0, \mathbf{R}), \\
p(\delta_t|\delta_{t-1}) &= \Pi^{\delta_t,\delta_{t-1}}, \\
r(\delta) &\sim p(r|\delta),
\end{aligned} \tag{2}
$$

where $\mathbf{h}_c(x_t, y_t)$ is the camera model that transforms real-world coordinates to image pixels, $\mathbf{y}_t$ is the measurement of position and wing flutter level obtained from the image, $z_t$ is a virtual measurement obtained as the radial distance in real-world coordinates $\sqrt{x_t^2 + y_t^2}$, $\mathbf{Q}_\delta$ and $\mathbf{R}$ are covariance matrices for the process noise and measurement noise respectively, $p_r(r|\delta)$ is a probability density function representing distribution of the expected radial distance given the mode, $\Pi^{\delta_t,\delta_{t-1}}$ is the transition probability matrix describing the probability of transitioning between modes, and $\mathcal{N}(\boldsymbol{\mu}, \boldsymbol{\Sigma})$ is the multivariate normal distribution with mean $\mu$ and covariance $\boldsymbol{\Sigma}$. The radial density $p_r(r|\delta)$ was modelled as

$$
p_r(r|\delta) = \begin{cases} C_s \cdot \mathcal{N}(r|0, \sigma_s^2) & 0 \leq r \leq R_o,\ \delta = s \\ C_f \cdot \mathcal{N}(r|R_o, \sigma_f^2) & 0 \leq r \leq R_o,\ \delta = f \\ 0 & \text{otherwise} \end{cases} \tag{3}
$$

where $C_s$ and $C_f$ are normalizing constants, $R_o$ is the outer radius of the funnel in real world coordinates and $\sigma_s^2$ and $\sigma_f^2$ are the respective variances.

The model is now complete and the objective is to estimate the mode. Since the model is non-linear and the number of possible mode transitions grows exponentially it is not straightforward to compute the optimal estimate, but a decent approximation is achieved using a filter bank of extended Kalman filters described in Gustafsson (2012).

For each sequence of modes $\delta_{1:t}$ the extended Kalman filter is applied in two steps for each iteration, the time update

$$
\hat{\mathbf{x}}_{t|t-1} = \hat{\mathbf{x}}_{t-1|t-1}, \tag{4a}
$$

$$
\mathbf{P}_{t|t-1} = \mathbf{P}_{t-1|t-1} + \mathbf{Q}_{\delta_t}, \tag{4b}
$$

and the measurement update

$$\begin{aligned}
\hat{\mathbf{y}}_t &= \mathbf{h}^{\delta_t}(\hat{\mathbf{x}}_{t|t-1}) && \text{(5a)}\\
\mathbf{S}_t &= \mathbf{H}_t \mathbf{P}_{t|t-1} \mathbf{H}_t^T + \mathbf{R}, && \text{(5b)}\\
\mathbf{K}_t &= \mathbf{P}_{t|t-1} \mathbf{H}_t^T \mathbf{S}_t^{-1}, && \text{(5c)}\\
\hat{\mathbf{x}}_{t|t} &= \hat{\mathbf{x}}_{t|t-1} + \mathbf{K}_t(\mathbf{y}_t - \hat{\mathbf{y}}_t), && \text{(5d)}\\
\mathbf{P}_{t|t} &= \mathbf{P}_{t|t-1} - \mathbf{K}_t \mathbf{H}_t \mathbf{P}_{t|t-1}, && \text{(5e)}\\
\mathbf{H}_t &= \nabla_{\mathbf{x}_t} \mathbf{h}^{\delta_t}(\mathbf{x}_t)|_{\mathbf{x}_t = \hat{\mathbf{x}}_{t|t-1}}. && \text{(5f)}
\end{aligned}$$

Note that there is an implicit dependence on $\delta_{1:t}$ for each variable that is not shown for clarity.

The likelihood of a mode sequence $\delta_{1:t}$, given the measurements, is computed as

$$\begin{aligned}
w_t^{\delta_{1:t}} &= p(\delta_{1:t}|\mathbf{y}_{1:t}, z_{1:t}) = \frac{p(\delta_{1:t})}{p(\mathbf{y}_{1:t}, z_{1:t})} p(\mathbf{y}_{1:t}, z_{1:t}|\delta_{1:t})\\
&= \frac{p(\delta_t|\delta_{t-1}) p(\delta_{1:t-1})}{p(\mathbf{y}_t, z_t|\mathbf{y}_{1:t-1}, z_{1:t-1}) p(\mathbf{y}_{1:t-1}, z_{1:t-1})}\\
&\qquad \times p(\mathbf{y}_t, z_t|\delta_{1:t}, \mathbf{y}_{1:t-1}, z_{1:t-1}) p(\mathbf{y}_{1:t-1}, z_{1:t-1}|\delta_{1:t})\\
&= \frac{p(\delta_{1:t-1})}{p(\mathbf{y}_{1:t-1}, z_{1:t-1})} p(\mathbf{y}_{1:t-1}, z_{1:t-1}|\delta_{1:t}) \cdot p(\delta_t|\delta_{t-1}) \qquad (6)\\
&\qquad \times \frac{p(\mathbf{y}_t, z_t|\delta_{1:t}, \mathbf{y}_{1:t-1}, z_{1:t-1})}{p(\mathbf{y}_t, z_t|\mathbf{y}_{1:t-1}, z_{1:t-1})}\\
&= w_{t-1}^{\delta_{1:t-1}} \Pi^{\delta_t, \delta_{t-1}} \frac{p(\mathbf{y}_t, z_t|\delta_{1:t}, \mathbf{y}_{1:t-1}, z_{1:t-1})}{p(\mathbf{y}_t, z_t|\mathbf{y}_{1:t-1}, z_{1:t-1})}\\
&\propto w_{t-1}^{\delta_{1:t}} \Pi^{\delta_t, \delta_{t-1}} p(\mathbf{y}_t, z_t|\delta_{1:t-1}, \mathbf{y}_{1:t-1}, z_{1:t-1}).
\end{aligned}$$

where the notation $A_{a:b} = \{A_a, A_{a+1}, \ldots, A_b\}$ has been used.

Assuming independence between $\mathbf{y}_{1:t}$ and $z_{1:t}$ conditional on the modes $\delta_{1:t}$, i.e. $p(\mathbf{y}_{1:t}, z_{1:t}|\delta_{1:t}) = p(\mathbf{y}_{1:t}|\delta_{1:t}) \cdot p(z_{1:t}|\delta_{1:t})$ and using the predicted measurement $\hat{\mathbf{y}}_t^{\delta_{1:t}}$ and its covariance $\mathbf{S}_t^{\delta_{1:t}}$ from the extended Kalman filter, (6) reduces to

$$w_t^{\delta_{1:t}} \propto w_{t-1}^{\delta_{1:t-1}} \Pi^{\delta_t, \delta_{t-1}} \mathcal{N}\big(\mathbf{y}_t; \hat{\mathbf{y}}_t^{\delta_{1:t}}, \mathbf{S}_t^{\delta_{1:t}}\big) p(z_t|\delta_t) \qquad (7)$$

To avoid combinatoric issues, pruning is applied to discard unlikely mode sequences, leaving a likely sequence of modes used as an estimate for when the bird is in *flying* or *stationary* mode. The corresponding position estimate $\hat{\mathbf{x}}_{t|t}^{\delta_{1:t}}$ for this sequence gives the filtered position of the bird in real-world coordinates.

The advantage of this approach is that the behaviour for the two modes of the bird, *flying* and *stationary*, can be modelled using various indicators from image processing, and an estimate of the mode is obtained by weighting these indicators rather than using deterministic rules. The parameters need to be determined for each setup and bird species, where some are determined physically from the setup, some can be estimated from historical behaviour of the species and some are tuned manually.

This approach works well for real-time applications, estimating the mode online, however it is possible to improve on the method by applying an offline algorithm. To increase robustness the estimated sequence is post-processed to filter out infeasible transitions, but this need is reduced if an offline algorithm is used instead and if the model is properly tuned.

# References


Chiranjeeb Buragohain and Subhash Suri. Quantiles on streams. In *Encyclopedia of Database Systems*, pages 2235–2240. 2009.

Michael Felsberg, Per-Erik Forssén, and Hanno Scharr. Channel smoothing: efficient robust smoothing of low-level signal features. *IEEE Transactions on Pattern Analysis and Machine Intelligence*, 28(2):209–222, Feb 2006.

Michael Felsberg, Kristoffer Öfjäll, and Reiner Lenz. Unbiased decoding of biologically motivated visual feature descriptors. *Frontiers in Robotics and AI*, 2(20), 2015.

Per-Erik Forssén. *Low and Medium Level Vision using Channel Representations.* PhD thesis, Linköping University, Sweden, SE-581 83 Linköping, Sweden, March 2004. Dissertation No. 858, ISBN 91-7373-876-X.

Fredrik Gustafsson. *Statistical Sensor Fusion.* Studentlitteratur, Lund, 2012. ISBN 9789144077321.
